# Feasibility and accuracy of using different methods to detect pregnancy by conceptus-stimulated genes in dairy cattle

**DOI:** 10.3168/jdsc.2020-0062

**Published:** 2021-03-26

**Authors:** Priscila Assis Ferraz, Carlos Alberto Souto Godoy Filho, Cecília Constantino Rocha, Adomar Laurindo Neto, Gabriela de Andrade Bruni, Thais Sayuri Imura Oshiro, Pietro Sampaio Baruselli, Fabio Soares Lima, Guilherme Pugliesi

**Affiliations:** 1 Department of Animal Reproduction, School of Veterinary Medicine and Animal Science, University of São Paulo, Pirassununga, São Paulo 00508-900, Brazil; 2 Department of Animal Science, University of Florida, Gainesville 32608; 3 Department of Population Health and Reproduction, School of Veterinary Medicine, University of California, Davis 95616

## Abstract

Development of new methods for early diagnosis of pregnancy can be important to increase the reproductive efficiency and profitability of dairy herds. The bovine conceptus secretes IFN-τ that stimulates the transcription of several genes in circulating immune cells and extrauterine tissues. The aims of this study were to evaluate the mRNA abundance for pregnancy predictability of a classic gene stimulated by IFN-τ (*ISG15*) and a novel potential pregnancy marker (*LGALS3BP*) in peripheral blood mononuclear cells (PBMC), total blood leukocytes (TBL) or milk leukocytes (TML), and cervical cells (CC) on d 20 after timed artificial insemination (TAI) in dairy cattle. Eighteen Holstein females (12 cows and 6 heifers) were submitted to an estrous synchronization protocol for TAI (d 0). On d 20 post-TAI, blood samples were collected from coccygeal vessels for isolation of PBMC and in Tempus Blood RNA tubes (Applied Biosystems) for TBL. Samples of CC were collected using a cytological brush, and the TML were isolated from milk samples collected before routine milking. Pregnancy diagnosis was performed on d 30 post-TAI using transrectal ultrasonography, and females were classified as pregnant (n = 8) or nonpregnant (n = 10). Total RNA was extracted and mRNA abundance of target genes (*ISG15* and *LGALS3BP*) was quantified by reverse-transcription quantitative PCR and normalized in relation to reference genes. Data were analyzed by ANOVA using the MIXED procedure of SAS (SAS Institute Inc.). The mRNA abundance of *ISG15* was greater in pregnant than in nonpregnant animals for PBMC, TBL, and CC. No difference was detected for TML based on pregnancy status. For *LGALS3BP* mRNA abundance, no difference was detected between pregnant and nonpregnant animals for PBMC, TBL, and TML, but a tendency for greater abundance in pregnant animals was observed for CC. The fold change for *ISG15* in each pregnant cow related to the mean of nonpregnant animals was 2.73 ± 0.31, 3.40 ± 2.17, 1.64 ± 0.29, and 0.005 ± 0.002 for PBMC, CC, TBL, and TML, respectively. The fold change for *LGALS3BP* in each pregnant cow related to the mean of nonpregnant animals was 0.97 ± 0.38, 1.77 ± 0.39, 0.20 ± 0.08, and 0.70 ± 0.11 for PBMC, CC, TBL, and TML, respectively. The receiver operating characteristic curve analysis indicated that *ISG15* abundance predicted pregnancy in PBMC (area under curve, AUC = 0.92) and CC (AUC = 0.77) but not in TBL (AUC = 0.72) or TML (AUC = 0.52). In conclusion, mRNA abundance for *ISG15* in PBMC was the best predictor for pregnancy at d 20 post-TAI, whereas TBL and TML were not good predictors of pregnancy on d 20 post-TAI. The mRNA abundance of *LGALS3BP* was not associated with pregnancy status in any type of cell evaluated.

## Highlights

• Peripheral blood mononuclear cells (PBMC) are the best biomarker for pregnancy prediction on d 20 after timed AI.
• An abundance of *ISG15* was observed in pregnant dairy females in PBMC, total blood leukocytes, and cervical cells.
• Abundance of *LGALS3BP* is not good biomarker for determination of pregnancy status using any cell type.
• Total milk leukocytes are not useful for detection of genes stimulated by early pregnancy.

## Graphical Abstract

**Figure:**
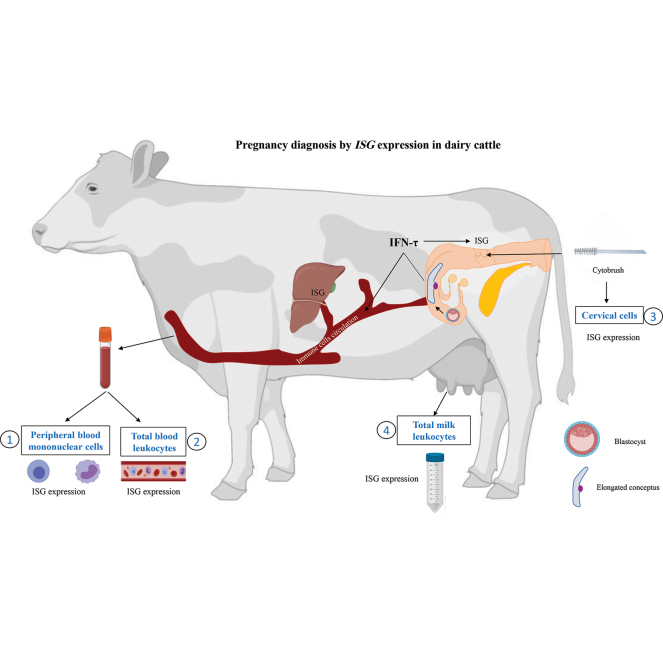
**Summary:** Interferon-tau (IFN-τ) is a pregnancy recognition signal secreted from the trophectoderm cells of the bovine conceptus that induces the expression of IFN-stimulated genes (ISG) in immune cells. The expression of ISG in peripheral blood leukocytes has been suggested as a potential tool for early pregnancy diagnosis. IFN-τ induces an increase in abundance of several ISG in peripheral mononuclear leukocytes between 18 and 20 days after fixed-time AI. We evaluated the abundance of 2 genes (*ISG15* and *LGALS3BP*) stimulated by the conceptus using samples collected from 4 cell types: peripheral blood mononuclear cells (PBMC), total blood leukocytes, cervical cells, and total milk leukocytes, and we compared these four methods as pregnancy predictors on day 20 after timed AI in dairy cattle.

Detection of nonpregnant cows is an important factor for reproductive performance in dairy herds, as it can have great effects on reproductive efficiency and economic gains in dairy production systems (Lucy et al., 2004). Novel methods for early pregnancy diagnosis in cattle have been investigated to detect non-pregnant cows on the third week of pregnancy (Spencer and Bazer, 2004; Hansen et al., 2017), decreasing the interval between timed AI (**TAI**) services (Ribeiro et al., 2012; Fricke et al., 2016). Although previous studies have indicated that determination of IFN-τ–stimulated genes (**ISG**) in circulating immune cells can be used as a potential pregnancy diagnostic test in beef (Matsuyama et al., 2012; Pugliesi et al., 2014; Melo et al., 2020) and dairy (Shirasuna et al., 2012; Haq et al., 2016) cattle, there is still no method with proven accuracy and feasibility for detection of pregnancy before d 20 of pregnancy (Green et al., 2010; Yoshino et al., 2018; Melo et al., 2020). Many studies have investigated ISG mRNA abundance using peripheral blood mononuclear cells (**PBMC**). Recently, other studies have used total immune cells from whole blood (Yoshino et al., 2018) or milk (Schanzenbach et al., 2017) or cervical and vaginal mucosa cells (Kunii et al., 2018) for accessing gene expression and predicting pregnancy status. In addition, novel potential pregnancy markers were recently reported by our group in beef heifers (Rocha et al., 2020) and need to be tested for early pregnancy diagnosis in dairy cattle. From these potential markers, *LGALS3BP* expression in immune cells was always 50% greater in pregnant heifers from d 10 to 20 of pregnancy. Previous studies described a possible role of this gene with the cellular adhesion process in the bovine endometrium during the preattachment period (Bauersachs et al., 2006; Okumu et al., 2011). Therefore, the aims of this study were to evaluate the mRNA abundance of a classic ISG (*ISG15*) and a novel potential pregnancy marker (*LGALS3BP*) as a predictor for pregnancy at d 20 post-TAI in dairy cattle using PBMC, total blood leukocytes (**TBL**), total milk leukocytes (**TML**), or cervical cells (**CC**). We hypothesized that the proximity between cervical and vaginal cells and the bovine conceptus location would induce greater mRNA abundance of pregnancy markers and hence better pregnancy predictability in CC cells than in circulating immune cells.

In the present study, lactating Holstein cows (7 multiparous and 5 primiparous) and 16-mo-old heifers (n = 6) with a BCS of 3.1 ± 0.3 (1-to-5 scale, Ferguson et al., 1994) maintained at the research farm in the Fernando Costa Campus, University of São Paulo (Pirassununga, SP, Brazil), were used. This study was approved by the Institutional Animal Care and Use Committee of the School of Veterinary Medicine and Animal Science of the University of São Paulo, São Paulo, Brazil (CEUA-FMVZ/USP 5490101219). The cows were milked twice daily and produced an average of 24.9 ± 4.7 kg of milk/d. Only cycling and mature heifers in good health and with absence of reproductive abnormalities were used. Cows were housed in freestall barns and heifers in grazing paddocks, and all animals were maintained with corn silage and received mineral salt and water ad libitum. All animals were subjected to an estradiol- and progesterone-based protocol for synchronization of ovulation and TAI according to the protocol described by Melo et al. (2020). On the day of TAI (d 0), animals were artificially inseminated by a single operator using frozen thawed semen. On d 20 after TAI, blood and milk (only cows) samples were collected and cervical cytology was performed for obtaining PBMC, TBL, TML, and CC samples. These 4 cell types were used to access the expression of potential pregnancy markers on d 20. Confirmatory pregnancy diagnosis was performed on d 30 using B-mode transrectal ultrasonography with a linear transducer (5 MHz, Myndray DP50 Vet) through the detection of a viable embryo with a heartbeat, and female animals were classified as pregnant (n = 5 cows and 3 heifers) or nonpregnant (n = 7 cows and 3 heifers).

Blood samples were collected from coccygeal vessels for PBMC (lymphocytes and monocytes) isolation using 10-mL sodium heparinized tubes (BD Life Sciences) and for TBL isolation using 9-mL Tempus Blood RNA tubes (Applied Biosystems). After collection and manual homogenization, the blood samples for PBMC isolation were immediately placed on ice and submitted to the isolation protocol right after animal handling. Blood samples for TBL isolation were immediately inverted 10 times and placed on ice until storage at −80°C before RNA extraction. Isolation of PBMC was performed by Ficoll (Ficoll-Paque Plus, GE Healthcare) gradient protocol according to Pugliesi et al. (2014).

The CC samples were obtained by cervical cytology using a cytological brush (Cytobrush, Viamed Ltd.) coupled to the tip of a conventional AI gun, covered by a disposable AI sheath and protected by a sanitary sheath, as described previously by Cardoso et al. (2017). The apparatus was inserted via the cervix and rotated to recover cells from the cervical canal near the external canal of the cervix. The cytobrush was then uncoupled from the apparatus and placed into a 2-mL cryotube filled with 1 mL of Trizol reagent (Life Technologies) and stored in liquid nitrogen at −96°C until mRNA extraction.

For isolation of TML, milk samples (300 mL) were collected from lactating cows during routine milking and stored in ice until processing. Milk was centrifuged and the fat layer was discarded, and the cell pellet was washed in 50 mL of cold PBS followed by centrifugation. The cell pellet was stabilized in 1 mL of Trizol Reagent (Life Technologies) and frozen at −80°C until RNA extraction, as described by Schanzenbach et al. (2017).

To check the immune cells in PBMC and TML, freshly isolated samples of each cell type were placed on a slice and stained with the fast panoptic dye method for morphological identification of cells by light microscopy under 400× magnification. Samples were considered pure when at least 95% of the 200 counted cells were lymphocytes and monocytes for PBMC samples and leukocytes for TML samples.

Isolation of RNA from TBL was performed using the Tempus Spin RNA Isolation Kit (Applied Biosystems) in accordance with the manufacturer's guidelines. After the samples were thawed at room temperature, the whole blood was transferred to 50-mL conic tubes, diluted in 3 mL of PBS, passed through the column, and submitted to consecutive centrifugations to wash the RNA. The RNA was eluted with 100 μL of DNase RNase free water. The RNA from samples of PBMC, TML, and CC was extracted using Trizol Reagent (Thermo Fisher Scientific) following the manufacturer's instructions. The concentration and purity of total RNA extracts were evaluated using a spectrophotometer (NanoVue, GE Healthcare).

The total RNA was treated with DNase I (DNase I Amplification Grade, Life Technologies). The RNA (700 ng) was submitted to reverse transcription (**RT**) reaction using a High-Capacity cDNA RT Kit (Life Technologies) according to the manufacturer's instructions. The cDNA was stored at −20°C until quantitative PCR (**qPCR**) analysis. Quantification of specific transcripts was performed by RT-qPCR using PowerUp SYBR Green Master Mix (Life Technologies), and reactions were carried out using a Step One Plus apparatus (Life Technologies). The selected target genes were a classic ISG, IFN-stimulated protein 15 kDa (*ISG15*), and a novel potential pregnancy marker, galectin-3 binding protein (*LGALS3BP*). The genes *GAPDH*, cyclophilin A (*PPIA*), ribosomal RNA (*18S*), and β-actin (*ACTB*) were tested as reference genes. Primers were previously described for cattle (Melo et al., 2020; Rocha et al., 2020).

The amplification data were extracted from the Step One Plus apparatus, and each sample was analyzed through LinReg PCR software for baseline correction, determination of qPCR efficiency, and cycle quantification values per sample. To select reference genes, NormFinder software was used. *GAPDH* and *PPIA* were selected as the best reference genes for PBMC, and *GAPDH* and *ACTB* were selected for TBL, TML, and CC. Expression of each target gene relative to the expression of the reference genes was normalized using the comparative Ct method (Pfaffl, 2001).

Statistical analyses were performed using SAS software (version 9.2, SAS Institute Inc.). Data that were not normally distributed according to Shapiro-Wilk test were transformed to natural logarithms or ranks. The transcript abundance was analyzed by ANOVA using the MIXED procedure. Fold change was calculated by the ratio between the gene expression of each pregnant animal and the averaged expression of nonpregnant female animals for each cell type. The least significant difference test was used for comparisons among cell types. Heifer or cow within group was included in the statistical model as a random effect for comparisons of pregnancy status in each cell type, but heifers were not included in analyses for TML.

The evaluation of abundance of a classic ISG (*ISG15*) and a novel potential pregnancy marker (*LGALS3BP*) in PBMC, TBL, TML, and CC on d 20 of pregnancy in dairy animals indicated a divergent stimulus of bovine conceptus on expression of these 2 genes and according to the cell type sampled. The *ISG15* abundance (Figure 1A) was greater in the pregnant group than in the nonpregnant group only for PBMC (*P* = 0.004), TBL (*P* = 0.04), and CC (*P* = 0.05). No difference (*P* = 0.58) in *ISG15* abundance in TML was detected between the pregnant and nonpregnant groups. The increased *ISG15* abundance in pregnant animals on d 20 was expected because this moment coincides with the peak of IFN-τ secretion by the bovine trophectoderm and is fundamental for maternal recognition of pregnancy (Hansen et al., 2017; Soumya et al., 2017). For *LGALS3BP* abundance (Figure 1B), no difference was detected between pregnant and nonpregnant groups in PBMC (*P* = 0.31), TBL (*P* = 0.43), and TML (*P* = 0.65), but a tendency of greater (*P* = 0.07) abundance in the pregnant group was observed for CC. Also, the fold change between *ISG15* abundance in pregnant and the mean of nonpregnant animals was compared among the 4 cells type (Figure 2A), and *ISG15* abundance had a greater (*P* < 0.05) fold change in PBMC, CC, and TBL compared with TML. Likewise, the fold change between *LGALS3BP* abundance in pregnant and the mean of nonpregnant females (Figure 2B) was greater (*P* < 0.05) in CC and PBMC compared with TBL and TML.

**Figure 1:**
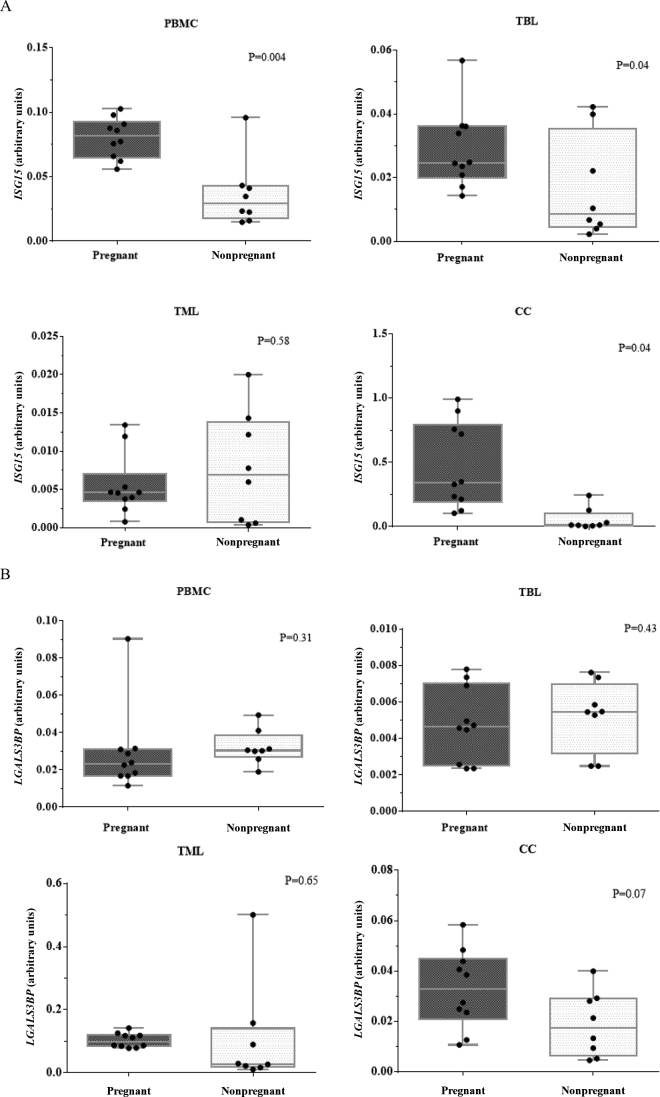
Box plots for the abundance of *ISG15* (A) and *LGALS3BP* (B) in peripheral blood mononuclear cells (PBMC), cervical cells (CC), total blood leukocytes (TBL), and total milk leukocytes (TML) on d 20 after timed AI in pregnant and nonpregnant dairy cows and heifers. The boxes show the interquartile range, means are indicated by continuous midlines, whiskers show the SEM, and dots show the individual values..

**Figure 2:**
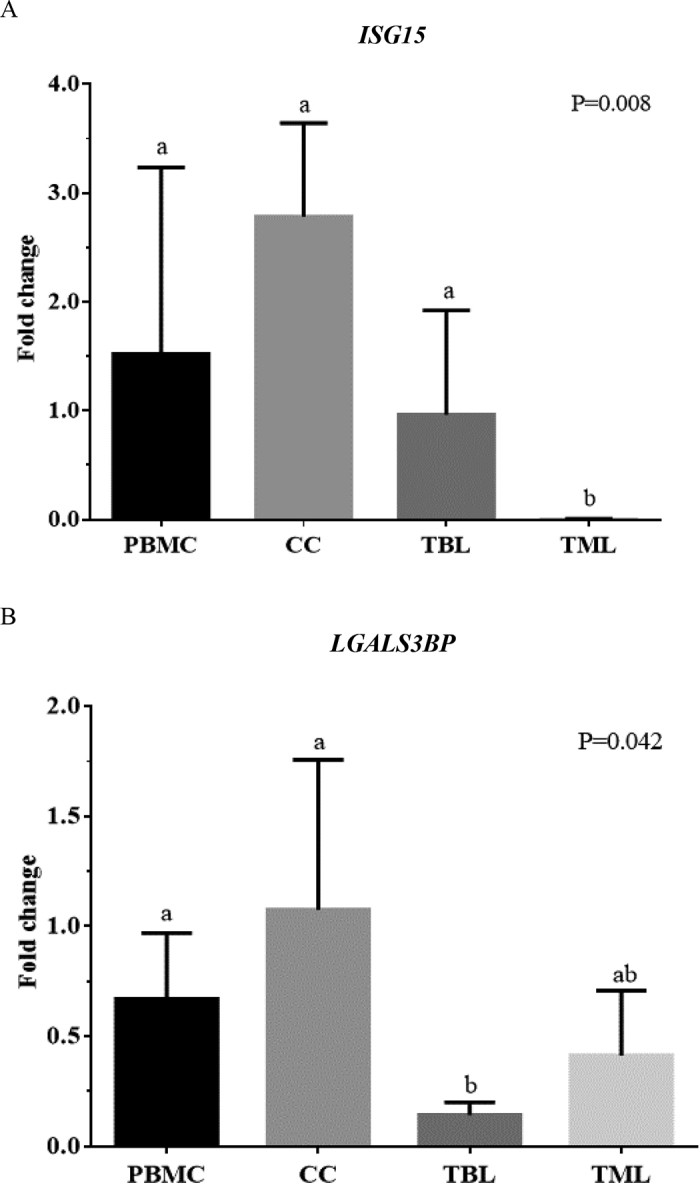
Fold change value of expression of *ISG15* (A) and *LGALS3BP* (B) between pregnant and nonpregnant dairy cows and heifers for peripheral blood mononuclear cells (PBMC), cervical cells (CC), total blood leukocytes (TBL), and total milk leukocytes (TML) 20 d after timed AI in dairy cows. Different letters (a, b) indicate significant differences between groups (*P* < 0.05).

The absence of stimulus on *ISG15* and *LGALS3BP* expression in TML of pregnant animals impaired the use of this cell type as a sample for pregnancy diagnosis based on ISG expression in lactating dairy cows. This nonexpression in immune cells presented in the milk may be a consequence of the pathways and diapedesis process for which immune cells are required for reaching the mammary alveolus. Also, the reduced fold change of *ISG15* in TML could be affected by parity order, as this cell type was evaluated only in lactating cows, and the response of ISG is more prominent and less variable in heifers than in cows (Green et al., 2010; Melo et al., 2020). Last, isolating TML is a time-consuming procedure for RNA expression analysis (Schanzenbach et al., 2017), which reduces the feasibility of using this as a method to diagnosis pregnancy in dairy cattle.

The extraction of mRNA from TBL without any isolation process would be an easier and faster method to determine the expression of pregnancy markers compared with the use of PBMC, as isolating the latter cells requires about 4 to 5 h. Also, several ISG have a similar profile in PBMC and granulocytes (Melo et al., 2020), which supports that accessing the TBL could indicate a similar increase in ISG expression in pregnant animals. Our findings supported that circulating immune cells from to TBL can also be sampled for determination of ISG expression, as the *ISG15* abundance in PBL was greater in pregnant animals and the fold change on its expression was similar compared with PBMC. However, for testing the hypothesis that expression of pregnancy markers in TBL is an efficient predictor of pregnancy in dairy cows, the present data were also analyzed using the MedCalc software package (version 19.1; Medcalc Software) to determinate the receiver operating characteristic (**ROC**) curve of each method using the *ISG15* expression as a predictor of pregnancy. The ROC curve analysis (Figure 3) indicated that *ISG15* abundance was a significant (*P* < 0.001) predictor of pregnancy in PBMC (AUC = 0.92) and CC (AUC = 0.77, *P* = 0.04) but not in TBL (AUC = 0.72, *P* = 0.15) or TML (AUC = 0.52, *P* = 1.00). Therefore, our hypothesis was not fully supported because the ROC curve analysis indicated a reduced AUC for TBL, and its sampling on d 20 was not a significant predictor of pregnancy on d 30. Similarly, Yoshino et al. (2018) reported that expression of ISG in granulocytes is more accurate compared with other methods using extracting RNA from whole blood for use as a pregnancy predictor in cattle. Therefore, although the use of whole blood for extraction of mRNA in circulating immune cells is simple and faster for applied use, this method results in reduced accuracy for pregnancy prediction and is still not feasible to replace the use of PBMC or granulocytes.

**Figure 3:**
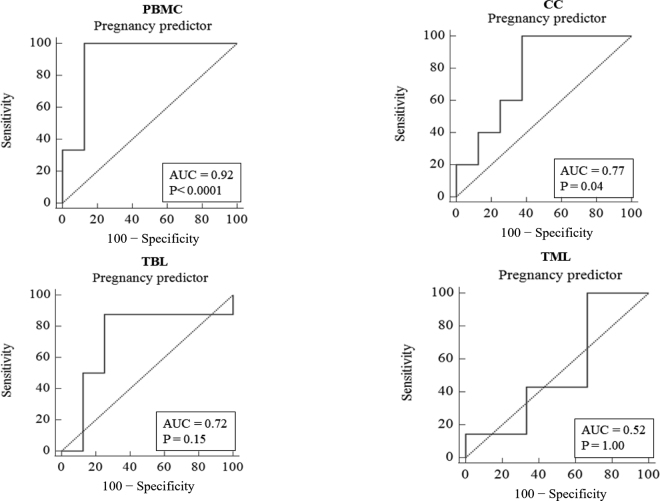
Receiver operator characteristic curves for peripheral blood mononuclear cells (PBMC), cervical cells (CC), total milk leukocytes (TML), and total blood leukocytes (TBL) from pregnant and nonpregnant dairy cows and heifers 20 d after timed AI. AUC = area under the curve.

Kunii et al. (2018) reported that *ISG15* expression in cervical and vaginal mucosal membranes of pregnant cows is significantly greater than that in nonpregnant cows. These authors also observed a greater fold change of *ISG15* expression in CC of pregnant animals, indicating that the closer location of the cervix in relation to the embryo may result in greater IFN-τ stimuli for ISG transcription. The present results did not fully support the hypothesis that the proximity between cervical and vaginal cells and conceptus location will induce greater expression of pregnancy markers in these cells than in circulating immune cells. The fold change in CC was similar between PMBC and TBL for *ISG15* expression in pregnant animals. For *LGALS3BP* expression, although the fold change in CC was similar to that in PBMC, it was greater than that in TBL, and a difference between pregnant and nonpregnant animals was observed only in CC. The relative expression of *LGALS3BP* and *ISG15* between pregnant and nonpregnant animals in vaginal cells was also recently evaluated by our group (Oshiro et al., 2020). However, in this study, no stimulus in mRNA abundance of these genes was observed in vaginal cells sampled by the cytobrush method on d 20 of pregnancy in beef heifers. Interestingly, in the results of the present study, a high coefficient of variation was observed within pregnant and nonpregnant groups, which resulted in a lower predictability in cattle, as shown by the reduced AUC for CC compared with PBMC.

The potential use of *LGALS3BP* expression as a pregnancy marker before d 20 post-TAI was recently indicated by our group because its expression is elevated in PBMC from d 10 to 20 of pregnancy in beef heifers (Rocha et al., 2020). Intriguingly, the present results were distinct, indicating that the presence of a viable bovine embryo did not stimulate *LGALS3BP* expression in immune cells from blood or milk but did stimulate it in CC samples. After the time of maternal recognition of pregnancy, the conceptus begins the processes of implantation that involve attachment and adhesion of the trophectoderm to the endometrial luminal epithelium (Spencer and Bazer, 2004). Its increased expression in this cell type is interesting because the involvement of *LGALS3BP* in the adhesion process of the conceptus is limited to the luminal epithelium of the uterus (Bauersachs et al., 2006), and, in addition to the different function of cervical and endometrial cells, they are not exposed to the same stimuli during pregnancy (Kunii et al., 2018). However, Baba et al. (2019) also observed that LGALS3BP modulates the maternal immune response during early pregnancy in buffaloes, suggesting that its higher expression at this period has an important role in maternal recognition of pregnancy and implantation.

In conclusion, the immune cells from milk are not stimulated by the presence of a bovine conceptus and are not a feasible sample for determining expression of genes stimulated by pregnancy. Although an approximately 2- to 3-fold increase in *ISG15* abundance is observed in TBL and CC of pregnant dairy animals, the determination of *ISG15* abundance using PBMC is the best pregnancy predictor on d 20 post-TAI among the cell types evaluated. In addition, the use of *LGALS3BP* abundance for determination of pregnancy status is not indicated for any sampling method.
